# Occurrence and potentially zoonotic genotypes of *Enterocytozoon bieneusi* in wild rhesus macaques (*Macaca mulatta*) living in Nanwan Monkey Island, Hainan, China: a public health concern

**DOI:** 10.1186/s12917-021-02916-8

**Published:** 2021-06-09

**Authors:** Wei Zhao, Huan-Huan Zhou, Guang-Xu Ren, Yu Qiang, Hui-Cong Huang, Gang Lu, Feng Tan

**Affiliations:** 1 Key Laboratory of Tropical Translational Medicine of Ministry of Education, Hainan Medical University, Haikou, 571199 China; 2 Department of Parasitology, Wenzhou Medical University, Wenzhou, 325035 Zhejiang China; 3 Qingdao Shinan District Center for Disease Control and Prevention, Qingdao, 266071 Shandong China; 4 Department of Pathogenic Biology, Hainan Medical University, Haikou, Hainan China; 5 Hainan Medical University-The University of Hong Kong Joint Laboratory of Tropical Infectious Diseases, Hainan Medical University, Haikou, Hainan China

**Keywords:** *Enterocytozoon bieneusi*, Rhesus macaques, Hainan, Zoonotic

## Abstract

**Background:**

*Enterocytozoon bieneusi*, a microsporidian species, is a zoonotic pathogen found in both humans and animals. Here, we determined the prevalence, explored the different genotypes of *E. bieneusi* in wild rhesus macaques (*Macaca mulatta*) (Hainan Island of China), and assessed their zoonotic potential.

**Methods:**

We collected 173 fecal specimens from wild rhesus macaques living in Nanwan Monkey Island, Hainan, China. Subsequently, we identified and genotyped *E. bieneusi* using nested PCR analysis amplification of the internal transcribed spacer region (ITS) of the rRNA gene. Lastly, a neighbor-joining tree was built based on gene sequences from the ITS region of *E*. *bieneusi*.

**Results:**

Of the 173 specimens from wild rhesus macaques, 26 (15%) were infected with *E. bieneusi*. We identified six genotypes of *E. bieneusi*, of which five were known: PigEBITS7 (*n* = 20), D (*n* = 2), Type IV (*n* = 1), Peru6 (n = 1), Henan-III (n = 1), and a novel genotype: HNM-IX (*n* = 1). From the phylogenetic analysis, the six genotypes identified here were all clustered into zoonotic group 1.

**Conclusion:**

This study is the first report to detect *E. bieneusi* infection in wild rhesus macaques from Hainan, China. Human-pathogenic genotypes D, Henan-III, Peru6, PigEbITS7, and Type IV in the wild rhesus macaques support these animals infected with *E. bieneusi* have a public health significance.

## Background

*Enterocytozoon bieneusi* is a typical human-pathogenic microsporidia species that invades the enterocytes of the small intestine [1]. Although its general infection is described by chronic diarrhea, and malabsorption or no clinical signs in immunocompetent humans, it can result in enhanced increased fatality via chronic diarrhea in individuals with immunodeficiency, such as patients with Acquired Immune Deficiency Syndrome (AIDS) [2]. Different studies have shown that it appears in several animals (mammals, birds, and reptiles) and some environmental samples (water, soil, and food) [3]. Most human infections result from the zoonotic transmission of spores through either contaminated food or water [4].

Recent surveys incorporated genotype information of *E. bieneusi* and elaborated genotype distribution among human populations and animal hosts [3]. Different studies have observed substantial genetic diversity within this species through sequencing the single internal transcribed spacer (ITS) region of the rRNA gene [5]. To date, scientists have detected approximately 600 *E. bieneusi* ITS genotypes [6]. Among these genotypes, 49 were found in both animals and humans [3]. All genotypes of *E. bieneusi* could be categorized into 13 clades [7]. Here, two large groups (1 and 2) that are composed of genotypes common in animals and humans are termed zoonotic. The remaining 11 groups (3 to 13) contain genotypes from specific hosts or wastewater [3]. Furthermore, *E. bieneusi* is commonly detected in various wildlife and a wide variety of both potentially host-adapted and zoonotic genotypes have been identified [3, 8]. Thus, wildlife is also an ecological resource for several human/animal infections. Therefore, the primary focus of epidemiological surveys should involve the genotyping of *E. bieneusi* isolates from under-sampled animal hosts with human contact to expand our knowledge regarding human microsporidiosis epidemiology and support *E. bieneusi* population analysis.

Rhesus macaques (*Macaca mulatta*) are prevalent in Southeast Asia, where their geographic range overlaps extensively with that of humans [9]. We carried out this study in the Nanwan Monkey Island, Nanwan peninsula, Lingshui county, south coast of Hainan, China. Globally, this is the only island-type nature reserve for rhesus macaques and is home to over 2500 monkeys. This island has a primitive natural environment, which makes it a perfect place for monkeys. Since its establishment in 1965, it has become a popular tourist destination. However, there is a lack of published studies on *E. bieneusi* infection in the rhesus macaques living in the Nanwan Monkey Island. Therefore, the aim of this study was to investigate the incidence and different genotypes of *E. bieneusi* present in the wild rhesus macaques.

## Results

### Infection rates of *E. bieneusi* in wild *M. mulatta*

Twenty-six of 173 specimens from wild rhesus macaques were positive for E. bieneusi since they amplified the ITS region of the rRNA gene, with an average infection rate of 15.0%. Besides, the infection rate of *E. bieneusi* in rhesus macaques less than 1 year of age (19.4%; 14/72) was higher than those animals older than 1 year (11.9%; 12/101) (Table 1). Meanwhile, out of all the positives, 14.2% (17/120) were females, and 17.0% (9/53) were males (Table 2). However, as illustrated in Tables 1 and 2, the infection rates difference were not statistically significant either by age or by gender.

**Table 1:** Prevalences of *E. bieneusi* and distributions of genotypes in *Macaca mulatta* by age

Age (Years)	Positive no./Examined no.(%)	Genotype(s) (n)	Statistics value
Less than one	14/72(19.4)	PigEbITS7(12); D(2)	χ^2^ = 1.88, *P* = 0.17
Over one	12/101(11.9)	PigITS7(8); Peru 6 (1); Type IV (1); HNM-IX (1); Henan-III (1)	
Total	26/173 (15.0)	PigITS7(12); D(2); Peru 6 (1); Henan-III (1); HNM-IX (1); Type IV (1)

**Table 2:** Prevalences of *E. bieneusi* and distributions of genotypes in *Macaca mulatta* by gender

Gender	Positive no./Examined no.(%)	Genotype(s) (n)	Statistics value
Male	9/53 (17.0)	PigITS7 (8); Type IV (1)	χ^2^ = 0.23, *P* = 0.63
Female	17/120 (14.2)	PigITS7(12); D(2); Peru 6 (1); HNM-IX (1); Henan-III (1)	
Total	26/173 (15.0)	PigITS7(12); D(2); Peru 6 (1); Henan-III (1); HNM-IX (1); Type IV (1)	

### Genotype distribution of *E. bieneusi* by gender and age

Six genotypes were identified in the wild rhesus macaques through sequencing and multiple sequence alignment. They included five known genotypes (D, Henan-III, Peru6, PigEbITS7, and Type IV) and one novel genotype (HNM-IX). Among them, genotype PigEbITS7 was dominant, and was found in 76.9% (20/26) of *E. bieneusi* isolates. All the remaining genotypes were at a lower frequency: 7.7% (2/26) for genotype D, and 3.8% (1/26) each for genotypes Peru 6, Type IV, Henan-III, and HNM-IX. Subsequently, two genotypes (D and PigEbITS7) were detected in the less than one-year-old animals, whereas five genotypes (Henan-III, HNM-IX, Peru 6, PigEbITS7 and Type IV) in the more than one-year-old animals (Table 1). As demonstrated in Table 2, two genotypes (PigEbITS7 and Type IV) were predominant in males, whereas five (D, Henan-III, HNM-IX, PigEbITS7, and Peru 6) in females.

### Genetic relationships of ITS genotypes

**Fig. 1:**
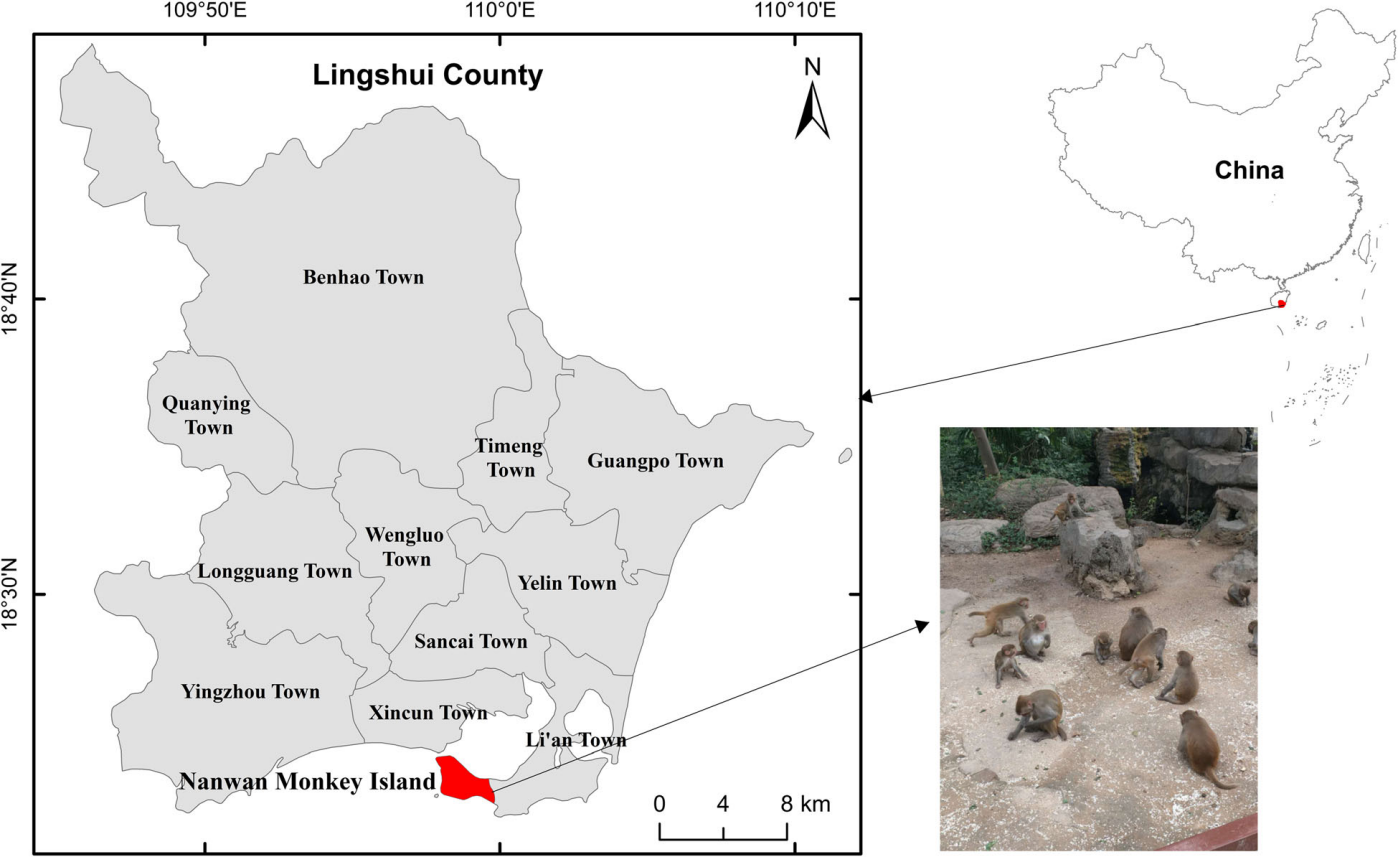
The location of the Nanwan Monkey Island, Hainan of China where the location of sample collection

Novel genotype HNM-IX had one single nucleotide polymorphism (SNP), with genotype EbpC (AF076042) having it at nucleotide site 51 of the ITS region. As illustrated in Fig. 1, phylogenetic analysis revealed that all genotypes belonged to zoonotic group 1. They were further sub-divided into different genotype sub-groups such as PigEbITS7 and D in subgroup1a; genotypes Peru 6 in subgroup 1b; genotype Type IV in subgroup 1c; and genotypes Henan-III and HNM-IX in subgroup 1d.

## Discussion

Non-human primates (NHPs) are known to possess a high genetic relationship with humans, which makes them useful biomedical research models. NHPs might be vulnerable to human diseases, thereby acting as zoonotic reservoirs, such as *Cryptosporidium*, *Giardia*, *Blastocystis*, etc. [9, 11]. In 1997, the first case of transference of *E. bieneusi* infection was recorded between a human (afflicted with AIDS) and a rhesus monkey (afflicted with simian immunodeficiency virus) [12]. However, until 2011, there was a lack of studies on the occurrence of *E. bieneusi* in non-human primates at the genotype level [11]. Zhao et al., summarized 16 studies on the infection of *E. bieneusi* in NHPs from seven countries [9, 13]. Among them, seven studies included rhesus macaques, and they were all from China, with a prevalence range from 4.2 to 31.1% [13–18]. For the first time, our study has detected *E. bieneusi* in wild rhesus macaques from the Hainan Province of China, with a prevalence of 15.0%. Generally, *E. bieneusi* has been found to be more prevalent in wild rhesus macaques here than other wild NHPs, such as baboons from Kenya (12.3%) [11], chimpanzees from Cameroon (4.5%) and Kenya (2.6%) [19], gorillas from the Central African Republic (4.0%) [20], orangutans from Indonesia (2.0%) [21], and five captive species of wild NHPs from the Qinling Mountains of China [17]. Our study showed that *E. bieneusi* was more prevalent in rhesus macaques than farm monkeys from Henan (6.8%), Guangxi (8.5%), Sichuan (10.5%), and zoo monkeys from Henan (12.5%) in China [15, 16, 22]. However, the prevalence of *E. bieneusi* in monkeys from Rwanda (18.0%) and some cities in China, like Shanxi (18.2%), Shanghai (26.7%), Hebei (27.0%), and Beijing (29.2%) was higher than that observed in our study [15, 16, 20, 22]. Additionally, there are two more studies that identified *E. bieneusi* infection in laboratory macaques in Beijing (25.6%) and Guangxi (18.5%), China, which were both higher compared with this study [14, 23]. In fact, in Hainan, two studies were reported on captive long-tailed macaques infected with *E. bieneusi,* which were also more than that observed in our study [9, 24]. Similar to humans and farm animals, age substantially increases the risk of *E. bieneusi* infection in NHPs [9]. Here, we identified a elevated *E. bieneusi* infection rate in young rhesus macaques compared with adults, which agreed with the results of captive long-tailed macaque and laboratory macaques from Hainan, China and North China, respectively [9, 15]. In addition to age, the health of the hosts, the detection methods, sample size, the experimental design, animal practices, etc. could cause the increase in prevalence.

Among the five known genotypes in our study, the genotype PigEbITS7 was detected in 76.9% (20/26) of *E. bieneusi* isolates, which shows predominance in the investigated wild rhesus macaques. This genotype was initially detected in pigs from the USA [8] and it has been confirmed to have a broad host range, even in humans [3]. In China, PigEBITS7 was detected in some patients, including AIDS and hospitalized children, and several animals such as rodents, NHPs, and urban wastewater [5, 9, 25–27]. Notably, genotype PigEBITS7 is also a common genotype in wild rodents such as asian house rats, brown rats and Chinese white-bellied rats, so this genotype may transmission from infected rodents to macaques [9].

Additionally, previous studies have reported the presence of four other genotypes (D, Henan-III, Peru6, and Type IV) in humans and animals around the world, of which genotypes D and Type IV are commonly found in *E. bieneusi*- induced microsporidiosis in humans [3, 28]. Both genotypes D and Type IV have been detected in infants, HIV-positive patients, and HIV-negative patients in China [25, 29–33]. Meanwhile, they have been found in NHPs, pigs, dogs, snakes, cats, hippopotamus, Pere David’s deer, chinchillas, Siberian tiger, lions, Fischer’s lovebird, red foxes, wastewater, and lake water [3]. In addition, genotype D has also recorded in other animals, such as bear, bornean orangutan, cattle, common crane, dog, goat, golden takin, golden snub-nosed monkey, hamadryas baboon, horse, rabbit, rat, raccoon, sheep [6]. Genotypes Peru 6 (syn. PtEbI, PtEbVII) (from Peru and Portugal) and Henan-III (from Malaysia and China) have been spread across limited geographical area as well as small number of *E. bieneusi*-infected human cases compared with genotypes D and Type I [34–36]. Meanwhile, genotype Peru 6 has been identified in sheep, goats, reindeers, and wastewater [37–40], whereas genotype Henan-III has been found in NHPs, pet snakes, pigs, and birds in China [41–44]. Therefore, the above shreds of evidence suggest the possible zoonotic transmission of these genotypes from the wild rhesus macaques to humans.

In this study, the novel genotype HNM-IX was genetically closely related to the human-pathogenic genotype EbpC which was commonly found in humans from Iran, Czech Republic, Peru, China, Thailand, and Vietnam [29, 45–48]. It was also found in more than 15 animal species and in environmental samples [3, 10]. From the phylogenetic analysis, the six genotypes identified here were all categorized into group 1. Group 1 had almost all human-pathogenic genotypes and possessed 94% of the known *E. bieneusi* ITS sequences [3]. Therefore, the genotypes in wild macaques investigated including the novel one could have a sizeable zoonotic possibility.

## Conclusions

This study is the first report to detect *E. bieneusi* infection in wild rhesus macaques from Hainan, China. Human-pathogenic genotypes D, Henan-III, Peru6, PigEbITS7, and Type IV in these animals support a zoonotic nature for *E. bieneusi*. Here, phylogenetic analysis showed that the novel genotype fell into group 1, suggesting its zoonotic possibility. Thus, visitors, veterinary workers, and the management of the wild rhesus macaques should be educated and informed to minimize the risk for transmission of *E. bieneusi* from those animals.

## Methods

### Fecal sample collection

**Fig. 2:**
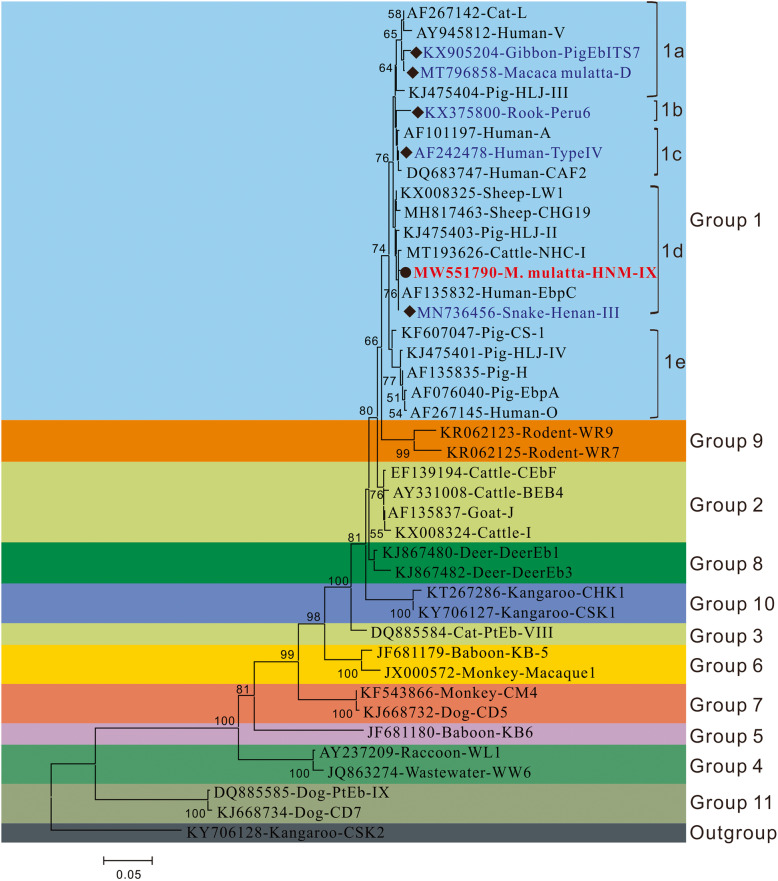
Phylogenetic relationship of *Enterocytozoon bieneusi* genotype groups. The relationship of *Enterocytozoon bieneusi* genotypes identified in the present study and other known genotypes deposited in the GenBank was inferred by neighbor-joining ITS sequences analysis based on the genetic distance using the Kimura two-parameter model. The numbers on the branches are percent bootstrap values from 1000 replicates. Each sequence is identified by its accession number, host origin, and genotype designation. The group terminology for the clusters is based on Zheng et al. [10]. The squares and circles filled in black indicate novel and known genotypes identified in this study, respectively

We obtained 173 stool samples from rhesus macaques in Nanwan Monkey Island, located on the Nanwan peninsula, Lingshui county, south coast of Hainan, in the southernmost province of China. It is geographically located at 109°48′ east longitude and 18°29′ north latitude (Fig. 2). In this study, the sampled macaques were from six major scenic areas of this island and approximately 30% of the total macaques of each areas were collected. Under the help of the trainer, we used sterile disposable latex gloves to collect fresh fecal samples, which were then placed in plastic cups marked with the collect date and the macaque’s age and sex. All specimens were transported to our laboratory and stored at 4 °C.

### DNA extraction

We sieved the fecal specimens, concentrated the filtrates, washed them thrice with distilled water, and centrifuged (10 min, 1500 g). Then, we used a QIAamp DNA Stool Mini Kit (Qiagen, Hilden, Germany) to extract genomic DNA from washed fecal specimens (180–200 mg) following manufacturer’s guidelines. Finally, DNA was eluted in 200 μL of AE buffer and stored at − 20 °C.

### PCR amplification

We analyzed all DNA preparations for *E. bieneusi* using nested PCR amplification. This amplification contained a nucleotide fragment (389 bp) containing 3′ end small subunit (SSU) (76 bp), ITS region (243 bp), and 5′ region of the large subunit (LSU) (70 bp) from *E. bieneusi* rRNA gene. Primers and cycle parameters were designed by Buckholt et al. [49]. Specifically, two pairs of primers, including EBITS3 and EBITS4 and EBITS1 and EBITS2.4, were used in the first and the second PCR amplification processes, producing nucleotide fragments of 435 and 389 bp, respectively. The two cycling parameters used for PCR reactions were as follows: 35 cycles of 94 °C for 30 s, 57 °C for 30 s, and 72 °C for 40 s, and 30 cycles of 94 °C for 30 s, 55 °C for 30 s, and 72 °C for 40 s, with both of them having a final extension step at 72 °C for 10 min. The TaKaRa Taq DNA Polymerase (TaKaRa Bio Inc., Tokyo, Japan) was used for all PCR amplifications. Meanwhile, all PCR amplification tests were carried out with positive controls (cattle-derived genotype BEB4 DNA) as well as a negative control (no DNA). Finally, all PCR products were separated via 1.5% agarose gel electrophoresis, followed by ethidium bromide staining.

### Nucleotide sequencing

All appropriately sized PCR products were purified using the Big Dye Terminator v3.1 Cycle Sequencing Kit (Applied Biosystems, USA) on an ABI PRISM 3730 XL DNA Analyzer (Sinogeno- max Biotechnology Co. Ltd., Beijing, China), followed by direct sequencing using PCR primers. We performed bidirectional sequencing to verify sequence accuracy.

### Sequence analysis

We determined *E. bieneusi* genotypes. Here, we aligned the nucleotide sequences with each other, and used the BLAST and Clustal X 1.83 to access the reference sequences. In particular, the first published names corresponded to the sequences who had a 100% resemblance to those from known genotypes. Otherwise, they were described as novel genotypes. Finally, the nomenclature was established by naming all genotypes according to the 243 bp of the ITS gene region of *E. bieneusi* [5].

### Phylogenetic analysis

Here, we studied the genetic association between the novel and known genotypes. Next, we used the Mega X software (http://www.megasoftware.net/) to compare the ITS region of all identified nucleotide sequences with those of the reference sequences. The neighbor-joining tree was built based on the evolutionary distances calculated using a Kimura 2-parameter model and bootstrap analysis of 1000 replicates.

## Data Availability

All data generated or analysed during this study are included in this published article. The identified nucleotide sequence of the novel genotype was submitted to the GenBank database (accession# MW551790).

## Abbreviations

AIDS: acquired immune deficiency syndrome
SNP: single nucleotide polymorphism
NHPs: non-human primates
BLAST: Basic Local Alignment Search Tool
ITS: internal transcribed spacer
SSU: small subunit
LSU: large subunit
